# Supplementary material for: Bio-inspired multimodal soft actuator with environmental self-adaptation
Source: Nat Commun. 2025 Aug 15;16:7630. doi: 10.1038/s41467-025-62328-2 (PMC12356950; doi:10.1038/s41467-025-62328-2)
Supplement: Supplementary file 1 — Supplementary Information [file 41467_2025_62328_MOESM1_ESM.pdf]

Supplementary Information for

Bio-inspired multimodal soft actuator with environmental self-adaptation

Chi Chen[1], Zixiao Liu[1], Pengju Shi[1], Yusen Zhao[1], Sidi Duan[1], Yingjie Du[1], Yichen Yan[1], Muqing Si[1], Tetsuya Iwasaki[2], Ximin He[1]*

Corresponding author: ximinhe@ucla.edu

The PDF file includes:
Materials and Methods
Supplementary Text
Supplementary Figures and Tables

Other Supplementary Material for this manuscript includes the following:
Supplementary Movie 1 to 15

1. Materials and Methods

1.1 Materials

Diacrylate liquid crystal monomers 2-Methyl-1,4-phenylene bis(4-(3-(acryloyloxy)propoxy)benzoate) (RM257) were purchased from Shijiazhuang Sdyano Fine Chemical, Co., Ltd. 2,2 -(Ethylenedioxy)diethanethiol (EDDET), pentaerythritol tetrakis(3-mercaptopropionate) (PETMP), I-2959, 1,6-Hexanedithiol (HDT), dipropylamine (DPA), tetraethyl orthosilicate (TEOS), ammonia solution (30%), poly(vinyl alcohol) (M_w 89,000-98,000) (PVA), and bis(2-ethylhexyl) sebacate (BEHS) were purchased from Sigma Aldrich. Sylgard 184 PDMS kits were purchased from Ellsworth. Dragon skin, a kind of silicone rubber, was obtained by Smooth On. Silicone glue (Permatex 82194), candles (Missyo), nitrile film (Comfy), tape (Scotch Magic), weighing paper (VWR International), PET film (Accutrex), PTFE tape (Anti-seize Technology), and candles were purchased from Amazon. PTFE membrane with 160 μm thickness used as the triboelectric positive material was purchased from Tisch Scientific (RS40316). All chemicals were used as received without further purification.

1.2 Materials preparation

Candle soot nanoparticles (CSNPs), serving as the photothermal agent, were collected by depositing them on aluminum foil held above a candle flame and subsequently scraping them off with a clean blade [1]. The preparation of LCE/CSNP followed our previous routine based on a two-step thiol-acrylate click reaction [2-4]. Specifically, 1,779 mg of RM257, 10.7 mg of I-2959, and 11.3 mg of CSNP were dissolved in 551.5 mg of toluene in a glass vial at 90 °C. Homogeneous dispersion of CSNP within the mixture was achieved via probe sonication for at least 3 min. Next, 96 mg of PETMP, 384 mg of HDT, and 340 μL diluted DPA solution (2 wt% in toluene) were added to the mixture at 90 °C under vigorous stirring. After 3 min of reaction, the mixture was cast into two tape-covered glass slides with a 280 μm spacer and cured at 60 °C overnight. The resulting LCE/CSNP film was then peeled from the mold and subjected to uniaxial stretching to 100% strain, followed by photocrosslinking under a UV lamp (UVGO, 365 nm) at an intensity of 35 mW/cm^2 for 2 hours on each side. As a result, a crosslinked LCE/CSNP monolayer with a final thickness of 140 μm was prepared for the subsequent assembly step.

The PDMS and Dragon skin film were prepared by mixing the base and curing agent at a ratio of 5:1. After vacuum defoaming for at least 5 min, the mixture was cast into glass molds with predefined spacers and cured at 80 °C overnight.

Thin platinum layers, serving as electrodes, were deposited by sputtering (Denton Desk II) on one side of the PDMS and on one side of the PTFE layer for 45 seconds and 60 seconds, respectively.

1.3 Fabrication of the asymmetric trilayer

The prepared LCE/CSNP and PDMS monolayer (Section 1.2) should be pretreated to enhance the adhesion before the assembly. Specifically, the LCE/CSNP film was cut into the size of 25 mm × 2.5 mm × 140 μm, followed by immersing in ethanol overnight. In the meantime, the PDMS film was cut into the size of 25 mm × 2.5 mm × 280 μm and washed with hexane. Both washed strips were treated with oxygen plasma for 3 min, followed by the deposition of a thin layer of silica coating using chemical vapor deposition with TEOS and ammonia solution for 90 min. Then, two LCE/CSNP strips and one PDMS strip were assembled using silicone glue (10 wt% in hexane) layer by layer from bottom to top (see Fig. S5A). It is important to note that during the assembly of the bottom layer of LCE/CSNP and PDMS, PTFE tape needed to be placed in the predefined position to block the glue and create the defect. Next, the assembled trilayer was transferred to an oven and heated at 60 °C for 3 days to remove hexane. After the PTFE tape was removed, the asymmetric trilayer with an unbound region was obtained (see Fig. S6). Unless otherwise specified, all tested trilayers have an X of 5 mm (Region I length) and a d of 3 mm (Region II length, same as the width of PTFE tape).

1.4 Fabrication of the triboelectric nanogenerator based on asymmetric structure

The triboelectric nanogenerator (TENG) was fabricated in a procedure similar to that of the asymmetric trilayer (Section 1.3), with the adaptation of replacing the inner PDMS layer with the combination of sputtered PDMS and PTFE layers (Section 1.2) (see Fig. S28). Therefore, the assembly sequence from bottom to top was modified to include the LCE/CSNP monolayer, sputtered PDMS layer, sputtered PTFE layer, and the other LCE/CSNP monolayer. In the meantime, the untreated PTFE tape, as the blocking layer, was still inserted between two sputtered layers to generate the vacancy for contact-separation mold.

1.5 Motion capture and analysis

All structures were mounted horizontally on a stage and illuminated by an 808 nm laser (LSR808NL, DPSS Laser) from a horizontal direction. The light spot had rectangular dimensions of 3.5 mm × 4.5 mm, which could fully cover the cross-sectional area of the actuator. Besides, a linear translational stage was fixed on the laser to control the precise height of the irradiation towards the sample. All motion was captured and recorded by a camera (EOS 70D, Canon) at 60 fps. The tips or the selected points were tracked by the software Tracker to measure angles and positions, which were subsequently used to calculate frequency and amplitude. Each point with different input was recorded for at least 5 s once the motion had stabilized, followed by analysis of the motion during every one-second period. The results were processed to obtain average frequencies and amplitudes.

Actuation under various wavelengths was performed using different light sources

including a red 635 nm laser (LSR635NL-100, DPSS Laser), a green 532 nm laser (Genesis MX-532, COHERENT), a blue 470 nm LED driver (SM1U25-A, Thorlabs) equipped with a collimator (M34 × 0.5 EXT, Thorlabs), and a solar simulator (HAL-320W, Asahi Spectra).

1.6 Thermomechanical characterizations

All samples were annealed at 150 °C for 5 min and then cooled to remove any thermal history. Moduli, stresses, and lengths were measured using a dynamic mechanical analyzer (DMA850, TA Instrument) under a 25 kPa preload, 1 Hz oscillation frequency, and a 5 °C/min heating rate. Isostrain mode was employed to measure internal stress and internal force when both ends were fixed without strain variation under 5 °C/min heating rate. The adhesion force was obtained by the peel testing, with two different layers fixed separately in clamps at each end.

1.7 Other characterizations

Differential scanning calorimetry (DSC250, TA Instrument) was performed to measure the nematic-to-isotropic transition of LCE/CSNP and the thermal property of PDMS. Specifically, 10 mg of the testing sample was sealed in an aluminum pan, which was heated to 200 °C at the rate of 10 °C/min and held for 2 min to erase the thermal history. Then, the sample was cooled to 0 °C at the rate of 10 °C/min, followed by the heating ramp to 200 °C again at the rate of 10 °C/min.

The cross-section morphology was captured by a scanning electron microscope (SEM) (Supra 40VP, ZEISS). The sample was sputtered with gold before the testing at 40 mA for 25 seconds by a sputter coater (Pelco SC-7). The CSNP morphology image was obtained using a scanning transmission electron microscope (TEM) (FEI Titan 80-300), with the CSNP dispersed in toluene and the solvent subsequently removed.

The photothermal process and thermographic images were captured by an infrared camera (TiX 580, Fluke) and analyzed by the software Fluke Connect. UV-Vis spectra were measured to investigate the absorption or transmittance using a spectrometer (UV-3101PC, Shimadzu). The particle size of CSNP was measured using dynamic light scattering (DLS) (Zetasizer Pro, Malvern Panalytical), with CSNP dispersed in toluene.

1.8 Setup for aquatic tests

The setup consisted of a tank to hold the sample stage and two NIR lasers to increase input energy, as heat dissipation underwater is significantly greater than in air (see Fig. S21A). The first laser was set horizontally facing the sample, while the second one was aligned at a ~15-degree angle to target the defect region. The PVA solution was prepared by dissolving the PVA powder in DI water with rapid stirring at 80 °C, followed by centrifugation in a mixer (ARE-310, Thinky) to remove the bubbles. Viscosity measurements were conducted by a rheometer (Discovery HR 30, TA

Instruments) at room temperature under the shear rate from 1 to 100 1/s. The tests under different water temperatures were based on a similar setup by tuning the temperature of the DI water.

1.9 Setup for cart design

Apart from a trilayer as the actuator, the setup consisted of a frame of Balsa wood and a PET film (see Fig. 4E). The mass of the actuator was 43.2 mg, while the total mass was 129.8 mg. The four feet of the frame were perforated to allow copper wires to pass through and guide the locomotion direction, which was directly opposite to the incident light. The cart was initially positioned away from the physical constraint to enable the free motion of the actuator. However, the distance between the copper wire and the constraint in the front path was less than the amplitude of the trilayer. For the physical contact demonstration, the light intensity to trigger the asymmetric trilayer was 3.967 W/cm^2, while the intensity for the symmetric trilayer was 6.723 W/cm^2. For the high-temperature passing demonstration, the light intensity was 4.772 W/cm^2. For the liquid-liquid interface passing demonstration, the upper layer was ethanol while the bottom layer was an ethanol solution with 5 wt% D-glucose and blue dye, and the light intensity was 9.348 W/cm^2.

1.10 Particle image velocimetry

First, the tracking particle, BEHS, was added to an atomizer for uniform dispersion in the air. After setting the stage and sample in a sealed acrylic chamber, a 532 nm laser (Genesis MX-532, COHERENT) equipped with a line pattern engineered diffuser was turned on to image the airflow (see Fig. S26A). The linear irradiation was aligned directly with the center of the sample. When no significant airflow can be observed, the NIR laser was activated to initiate the motion. The entire process was captured by a high-speed camera (Phantom with the 100 mm lens, Laowa) and analyzed by the PIVlab application in Matlab.

1.11 Triboelectric nanogenerator test

The assembled photothermal TENGs were tested by an electrochemical workstation (CH Instruments, CHI660E). The sputtered electrodes were connected to the working and counter electrodes of the electrochemical workstation, while the reference electrode was grounded. The open-circuit voltage of the photothermal TENGs was measured using the open-circuit potential-time mode of the electrochemical workstation. The entire setup was placed in a high-temperature oven equipped with a thermocouple to evaluate the output voltage under different temperature conditions. The peak voltage (V_{peak}) and average voltage (V_{avg}) were calculated using the following equations:

$$V_{\mathrm{peak}} = \max |V(t)|$$

$$V_{avg} = \frac{1}{T}\int_{t0}^{t0+T} V(t)dt$$

2. Supplementary Text

2.1 Development of the simulation model

This analytical model investigated the bifurcation criterion between oscillations and undulations. The top LCE/CSNP layer, the middle PDMS layer, and the bottom LCE/CSNP layer were denoted as layer 1, layer 2, and layer 3, respectively. Since the thermal responsiveness of the PDMS is several orders of magnitude lower than that of the LCE/CSNP (see Fig. S7B), it was assumed that the coefficient of thermal expansion of layer 2 is zero (α_2=0). The unbonded region is located at the interface between layer 2 and layer 3. For all remaining bonded regions, since no structural delamination was observed in experiments, their interfacial bonding can be assumed to be perfect, which suggests that PDMS and LCE/CSNP have the same strain at their interfaces. However, this relationship does not hold for the unbonded region.

The thermomechanical deformation process can be described as follows. First, the deviation of temperature T from the strain reference temperature T_{ref} leads to the generation of a thermal strain ε_{th}, governed by $\varepsilon_{th}=\alpha(T-T_{ref})$. For LCE/CSNP, α is negative. Then, axial elastic forces P_i (i=1, 2, 3) are generated due to the stiffness and strain mismatch between the different layers. Eventually, the structural asymmetry and excitation asymmetry lead to the bending behavior of the whole structure. It is assumed that plane sections remain plane and perpendicular to the curved axis when bending occurs. In summary, the total strain in each layer is contributed by thermal strain, axial strain, and bending strain.

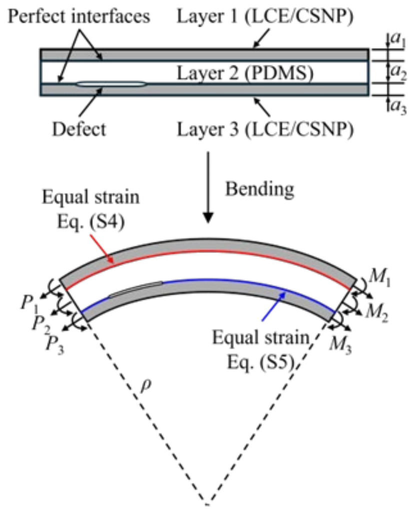

Fig. S1: Free body diagram of the composite structure with an artificial defect.

Assume that the boundary effect is neglected and only regions far away from the free end are considered. This assumption is reasonable because the external laser source mainly illuminates Region I, which has the largest deformation and the greatest

contribution to locomotion. Since there is no external force acting in these regions, all internal forces and moments must be in equilibrium, which can be expressed by Eqns. (S1)-(S2). The free body diagram of the structure is shown in Fig. S1.

$$P_1 + P_2 + P_3 = 0 \tag{S1}$$

$$\sum_{i=1}^{3} M_i - \frac{P_1 a_1}{2} - P_2\left(a_1 + \frac{a_2}{2}\right) - P_3\left(a_1 + a_2 + \frac{a_3}{2}\right) = 0 \tag{S2}$$

where M_i and a_i are the bending moments and thickness of layer i, respectively. The relationship between M_i and the radius of curvature ρ is given by

$$\frac{1}{\rho} = \frac{M_1}{E_1 I_1} = \frac{M_2}{E_2 I_2} = \frac{M_3}{E_3 I_3} = \sum_{i=1}^{3} M_i \Big/ \sum_{i=1}^{3} E_i I_i \tag{S3}$$

For the interface between layer 1 and layer 2, where the materials are considered to be perfectly bonded, the layer 1 and layer 2 have equal strains at their interface, mathematically expressed as

$$\alpha_1 \Delta T_{12} + \frac{P_1}{a_1 E_1} - \frac{a_1}{2\rho} = \frac{P_2}{a_2 E_2} + \frac{a_2}{2\rho} \tag{S4}$$

where ΔT_{12} is the temperature change relative to room temperature at this interface. On the left side of Eqn. (S4), the first term is the thermal strain, the second term is contributed by the axial force, and the third term is the bending strain.

The qualitative difference between oscillation and undulation is the direction of bending around the initial time. For oscillation, the top surface of the cantilever is illuminated and heated (because of the presence of gravity), and the entire structure directly bends up. For undulation, however, the top surface is still illuminated, but the structure bends down. It is well known that the bending direction is directly related to the strain gradient in the structure. If the layers are bonded without any artificial defect, the top surface will contract more than the bottom surface because the top surface will be at a higher temperature. However, the introduction of the artificial defect indicates that the geometric and stiffness constraints are partially removed so that the bottom surface is possible to contract more than the top surface, even though its temperature is lower. (Fig. 2B) This accounts for downward bending and undulation that does not occur in fully bonded structures. For the bifurcation point between oscillation and undulation, a strain equivalence at the bonded regions of the interface between layer 2 and layer 3 can be used as a bifurcation criterion.

$$\frac{P_2}{a_2 E_2} - \frac{a_2}{2\rho} = \alpha_3 \Delta T_{23} + \frac{L_0 - d}{L_0}\frac{P_3}{a_3 E_3} + \frac{a_3}{2\rho} \tag{S5}$$

The left and right sides of Eqn. (S5) are the strains of layer 2 and layer 3 at their interface, respectively. ΔT_{23} is the interfacial temperature change. The factor $(L_0\text{-}d)/L_0$ is the ratio of the length of the bonded region to the entire length. Only the bonded region has the axial strain P_3/a_3E_3, which results from the interaction between layers.

2.2 Development of the governing equation

The critical ratio K_0 between ΔT_{23} and ΔT_{12} [Eq. (S6)] delineates the transition between bending (oscillation) and buckling (undulation), which can be obtained by solving the equilibrium and strain compatibility conditions embodied in Eqns. (S1)-(S5).

$$K_0 = \frac{\Delta T_{23}^{\text{analy}}}{\Delta T_{12}^{\text{analy}}} = \frac{a_1 E_1 \left(a_1 a_2 E_2 L_0 + a_2^2 E_2 L_0 - a_1 a_2 E_2 d_0 - a_2^2 E_2 d_0 + a_1 a_3 E_3 L_0 + 2a_2 a_3 E_3 L_0 + a_3^2 E_3 L_0 \right)}{a_3 E_3 L_0 \left(a_1^2 E_1 + 2a_1 a_2 E_1 + a_1 a_3 E_1 + a_2^2 E_2 + a_2 a_3 E_2 \right)} \quad \text{(S6)}$$

Experiments can also provide a ratio K with the same definition $\Delta T_{23}^{\text{exp}}/\Delta T_{12}^{\text{exp}}$, while the interfacial temperature changes, ΔT_{12} and ΔT_{23}, can be measured directly using IR camera. When the K is greater than the theoretical ratio K_0, which suggests that layer 3 contracts more than layer 2, the whole structure bends down and undulates. Conversely, if $K<K_0$, the structure bends up and oscillates.

Due to the limited resolution of IR camera, it is difficult to obtain the exact temperature at the interfaces. Therefore, thermomechanical numerical simulations based on COMSOL Multiphysics were performed to observe the temperature changes during motions and obtain $\Delta T_{12}^{\text{exp}}$ and $\Delta T_{23}^{\text{exp}}$. Strains and stresses were simulated by Solid Mechanics module, and the temperature distributions were modeled in Heat Transfer in Solids interface. A multiphysics interface, Thermal Expansion, was employed to apply thermal strain to the structure, which is the link between thermal variable T and mechanical variable ε_{th}.

2.3 Energy output of different modes

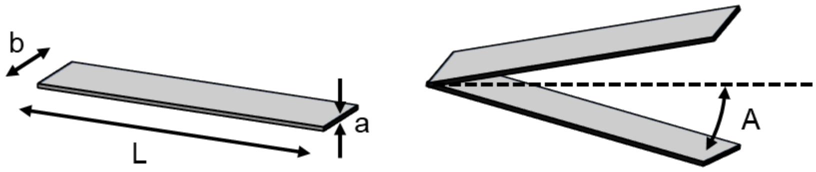

Fig. S2: Schematic diagram of oscillating trilayer.

Consider a cantilever beam with density ρ, length L, and width b, and thickness a. Its moment of inertia can be expressed as

$$J = \int_0^l \rho a b x^2 dx = \frac{1}{3}\rho a b L^3 \quad \text{(S7)}$$

The kinetic energy of the cantilever during oscillation can be estimated by

$$E_{\text{k,osci}}(t) = \frac{1}{2} J \dot{\theta}(t)^2 \quad \text{(S8)}$$

where θ is the angle of oscillation. For harmonic oscillations, θ can be expressed as a series of sinusoidal functions, i.e., $\theta(t)=\sum A_i \sin(\omega_i t)$, where the amplitudes A_i and frequencies ω_i can be determined by the experimental result of $\theta(t)$. The kinetic energy during oscillation can be given by

$$E_{\text{k,osci}}(t) = \frac{1}{2} J \dot{\theta}(t)^2 = \frac{1}{6}\rho a b L^3 \left[\sum A_i \omega_i \cos(\omega_i t) \right]^2 \quad \text{(S9)}$$

The kinetic energy of the cantilever beam during undulation cannot be estimated by the above method due to the complex mode shapes and anharmonic nature of the motion. Therefore, its kinetic energy has to be obtained by finite element simulations. The coordinates (x, y, z) and velocity v(x, y, z, t) of each point in the continuous structure at each moment can be obtained numerically by simulations. Therefore, the kinetic energy during undulation can be evaluated by the following integral.

$$E_{\mathrm{k,undu}}(t) = \int_V \frac{1}{2}\rho\left[v(x,y,z,t)\right]^2 dV \tag{S10}$$

3. Supplementary figures (Fig. S3 to S33) and tables (Table S1 to S3)

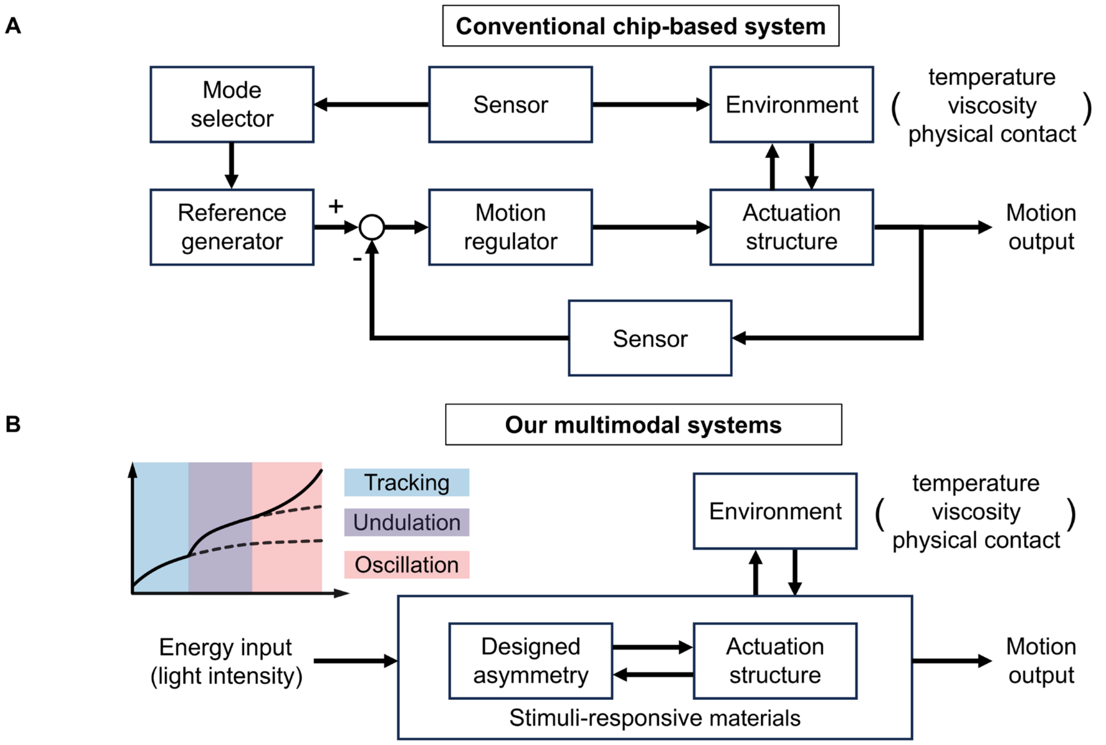

Fig. S3: Two distinct ways for the development of environment-adaptive multimodal actuators. (A) Conventional chip-based systems utilize computational intelligence in a hierarchical structure with multiple components: the mode selector determines the desired mode (*e.g.*, tracking, undulation, oscillation) based on the environment data detected through sensors; the reference generator specifies the desired motion in accordance with the selected mode; the motion regulator actuates the physical system (actuation structure) to align actual motion with the desired motion. (B) Physically intelligent systems embed the sensing-decision-actuation cycle directly within stimuli-responsive material systems via built-in feedback control.

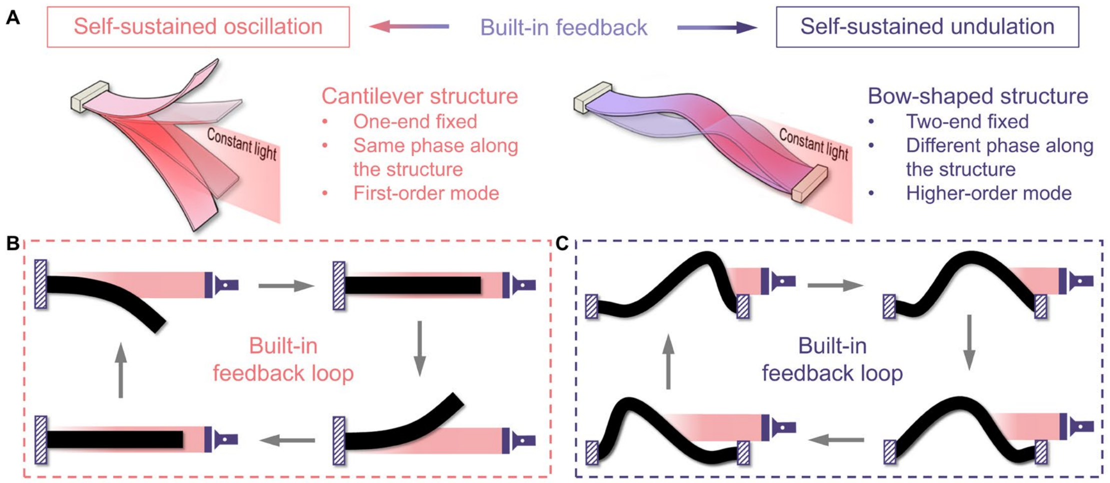

Fig. S4: Two self-sustained motion modes and their built-in feedback loops, presenting distinct characteristics. (A) Two common self-sustained motion modes are oscillation and undulation with different phases along the structure and different order modes, which are achieved by cantilever and bow-shaped structures, respectively. (B) The built-in feedback loop for self-sustained oscillation: Initially, an external force is introduced to disrupt the equilibrium (*i.e.*, a downstroke), causing localized illumination to heat the top surface of the photothermal cantilever structure above its transition temperature, resulting in the local shrinkage and upward bending. Due to the inertia, the tip of the structure overbends, thereby blocking the illumination and enabling the shrunk area will cool down and recover. Meanwhile, the opposite surface is illuminated, resulting in a downward stroke to complete the feedback loop. (C) The built-in feedback loop for self-sustained undulation: When the bow-shaped structure is exposed to horizontal light, its top surface contracts, forming a bump that moves away from the light until it arrests at the left clamped end, where a new crest forms at the right side. As the new crest grows, it shadows and eliminates the old crest through a pop-through transition, thereby repeating the process and completing the built-in feedback loop for self-sustained undulation.

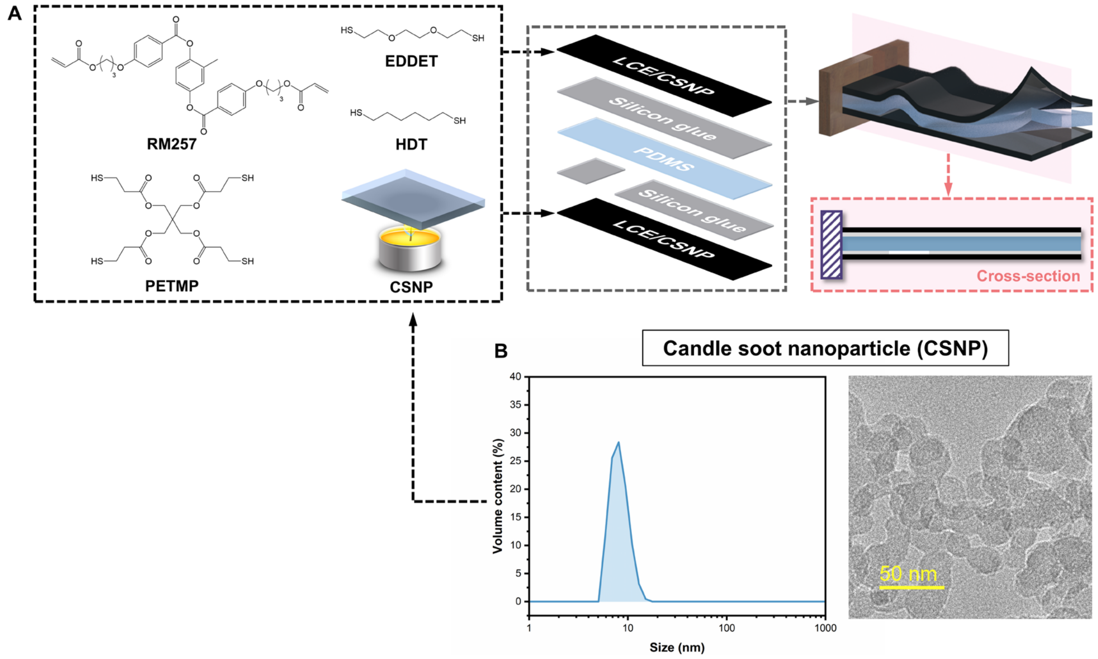

Fig. S5: Schematic illustration for the preparation of the asymmetric trilayer structure. (A) The whole structure was based on a symmetrical active-passive-active trilayer structure with an artificially introduced defect by partially blocking the silicon glue (see Section 1.3). (B) The active layers were composed of LCE with candle soot nanoparticles (CSNPs) that were dispersed as the photothermal agent (see Section 1.2). Dynamic light scattering (DLS) analysis revealed an average CSNP size of approximately 8.31 nm, consistent with the TEM image.

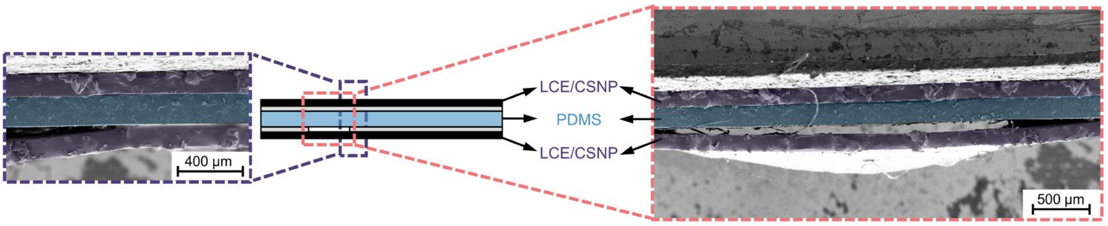

Fig. S6: Cross-section SEM images of the asymmetric trilayer structure. Three layers were glued completely with uniform thickness. In the meantime, the LCE/CSNP monolayer in the defect region blocked by the PTFE tape could move flexibly and exhibit different thermomechanical behavior from the bilayer.

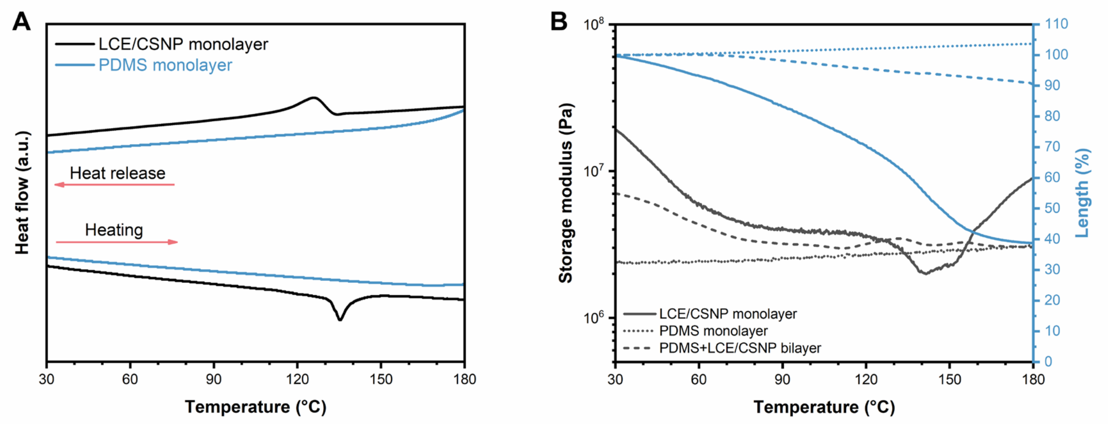

Fig. S7: Thermal analysis results. (A) DSC results presented the nematic-to-isotropic temperature (T_{ni}) of LCE/CSNP around 135 ºC, while no phase transition in PDMS monolayer. (B) The LCE/CSNP monolayer demonstrated large changes in modulus and length, while the PDMS was relatively stable under varying temperatures in the DMA test. The bilayer showed intermediate thermomechanical properties at working temperatures below ~ 120 ºC since the LCE/CSNP actuating layer had to sacrifice partial contraction to drive the passive PDMS layer to move synchronously.

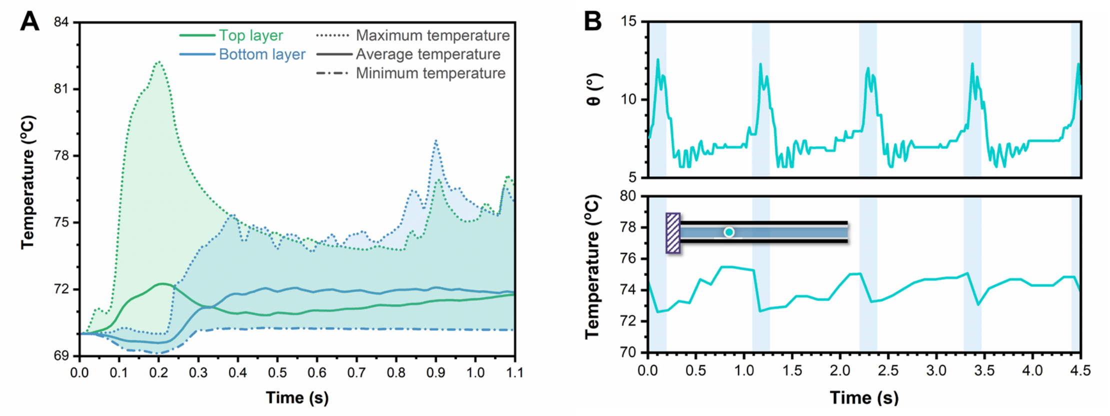

Fig. S8: Temperature variation in the instability-induced undulation cycle. (A) As simulation results suggest, for each cycle, the top layer has a higher temperature than the bottom layer during the buckling since it is heated directly. During the damping, the two temperature differences decrease to thermal equilibrium. (B) As analyzed from IR camera data (capturing the maximum temperature of the trilayer), buckling caused a rapid temperature drop due to strong convection cooling, followed by the excitation of Region III into oscillation via the self-shadowing mechanism. Over time, as damping weakened and oscillation speed decreased, the resulting reduction in convection cooling led to a gradual increase in overall temperature.

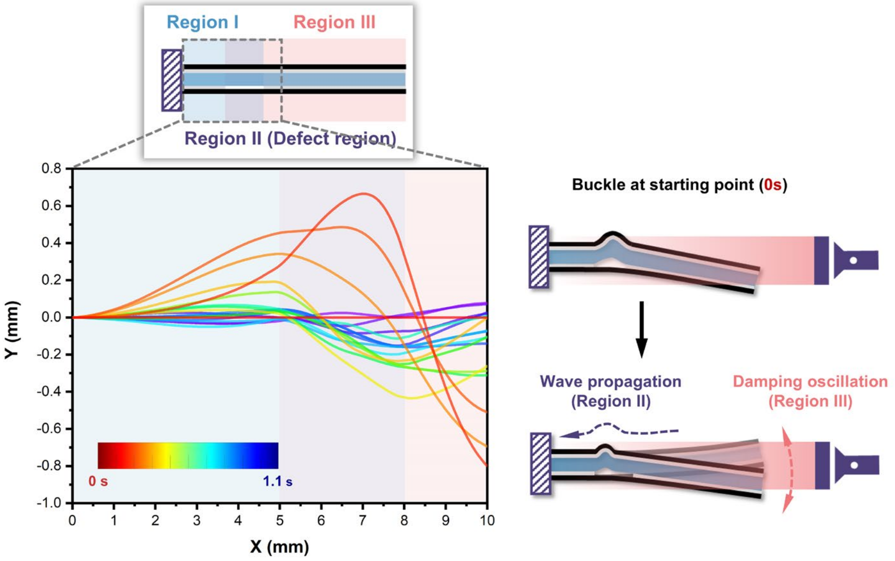

Fig. S9: The simulation results of shape morphing during the undulation process. One end of the trilayer was fixed (at X=0 and Y=0) horizontally and triggered by the irradiation from the right side. Region I, Region II (defect region), and Region III were located in the range of 0-5, 5-8, and >8 mm, respectively.

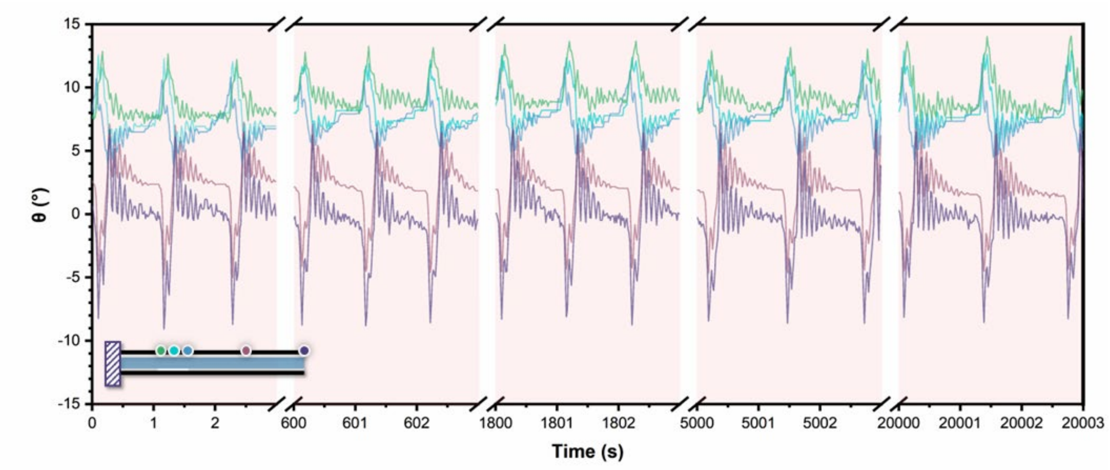

Fig. S10: Motion tracking with the angle change from the initial state for selected points over a long time period. Green, cyan, and blue refer to the left, middle, and right points of the defect region, while violet and purple refer to midpoint and tip of Region III, respectively. The process was triggered by the constant laser at 3.728 W/cm^2.

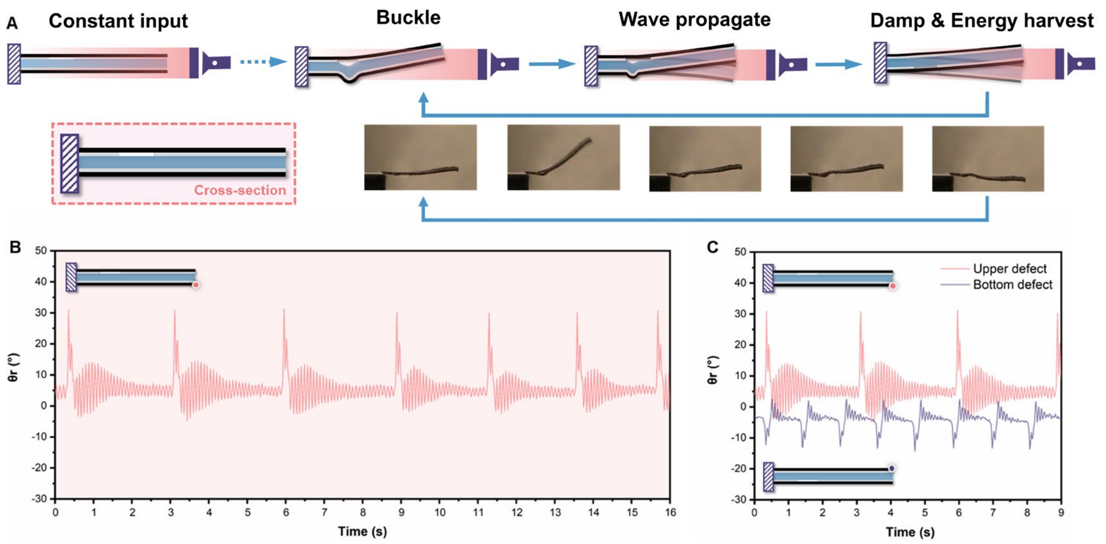

Fig. S11: The instability-induced undulation motion with the asymmetry in the upper half. (A) The fundamental mechanism was the same, based on the asymmetric structure, in generating the difference of internal stress for buckling. Since the monolayer LCE/CSNP was on the top while the bilayer was on the bottom, the buckling would result in upward bending first. Afterward, exposed to constant irradiation, the buckled defect region shrank and bent downward to power the oscillation of Region III. With the same direction of wave propagation along the light, Region III would harvest the energy for the next buckling to complete the cycle. (B) Motion tracking for the tip at the input of 3.728 W/cm^2. (C) Comparison of undulation behaviors between asymmetric trilayers with defects in different positions at the input of 3.728 W/cm^2. The oscillation frequencies were similar (12.416 Hz for the upper defect trilayer and 12.401 Hz for the bottom defect trilayer). However, the undulation frequencies exhibit a distinct difference (0.391 Hz for the upper defect trilayer and 0.729 Hz for the bottom defect trilayer), which might be related to gravity and suggests the upper counterpart needs to accumulate more energy to buckle.

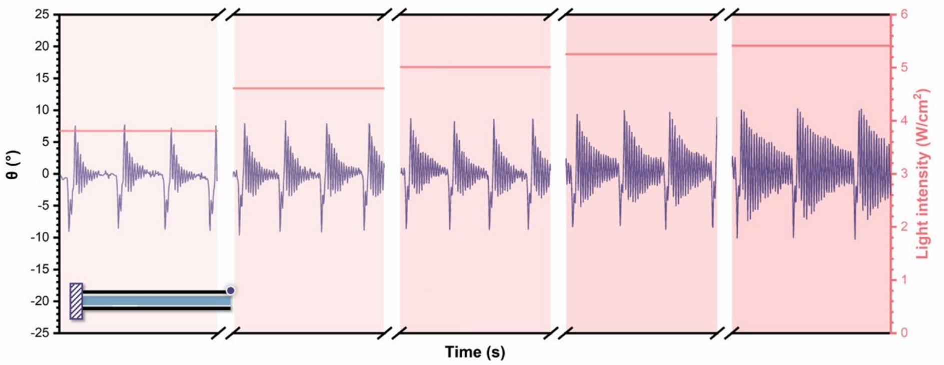

Fig. S12: The motion adjustment to the increasing input before the undulation-oscillation bifurcation point. With the increasing light intensity, the undulation frequency decreased gradually. Meanwhile, the residual amplitude increased rapidly with the maximum amplitude rising slowly.

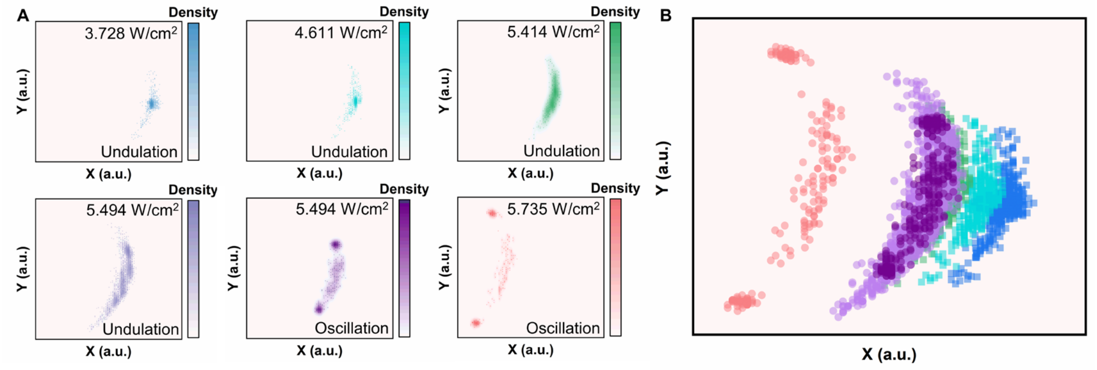

Fig. S13: The trajectory of the tip tuning by increasing input. (A) A distinct difference existed between the trajectories of undulation and oscillation in terms of the density distribution. Specifically, in undulation motion, the trajectory was concentrated in the middle area, while the highest density occurred at the two ends in oscillation motion, where the velocity of the tip decreased to 0. (B) The overall variation of the trajectory across the undulation to the oscillation. As the input enhanced, the trilayer would shrink accordingly, so the tips tended to move toward the stage. The amplitude exhibited a general trend of increasing amplitude. During the undulation-to-oscillation transition at the input of 5.494 W/cm^2, the two motions demonstrated a similar movement range with different characteristic density distributions, and the amplitude would drop slightly during the transition (see Fig. 2E).

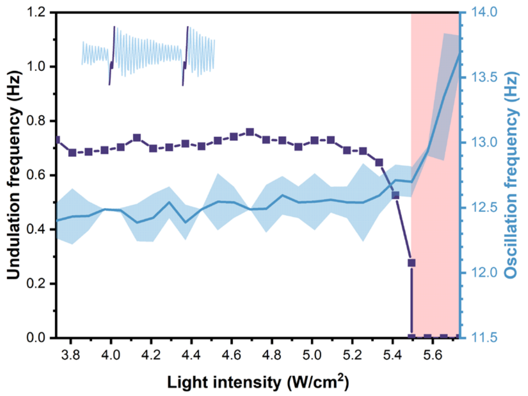

Fig. S14: The frequency adjustment to the increasing input. The undulation frequency was the same as the buckling frequency. In the undulation state, the oscillation frequency was the same as the damping frequency. After undulation-oscillation bifurcation, the undulation frequency vanished.

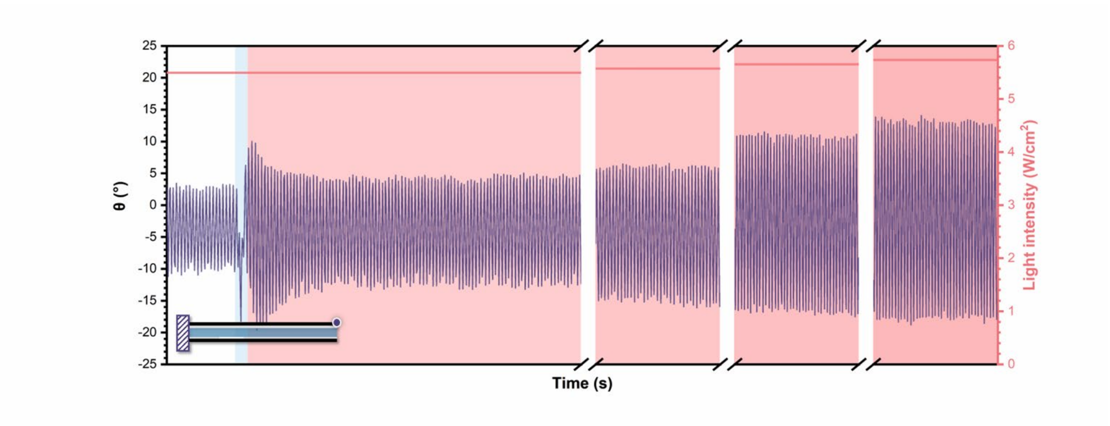

Fig. S15: The motion adjustment to the increasing input since the undulation-oscillation bifurcation. When the undulation-oscillation bifurcation occurred at the input of 5.494 W/cm^2, the trilayer would be locked in oscillation without any buckling. The only change was the enhancement of the amplitude and frequency (see Fig. 2E and Fig. S14).

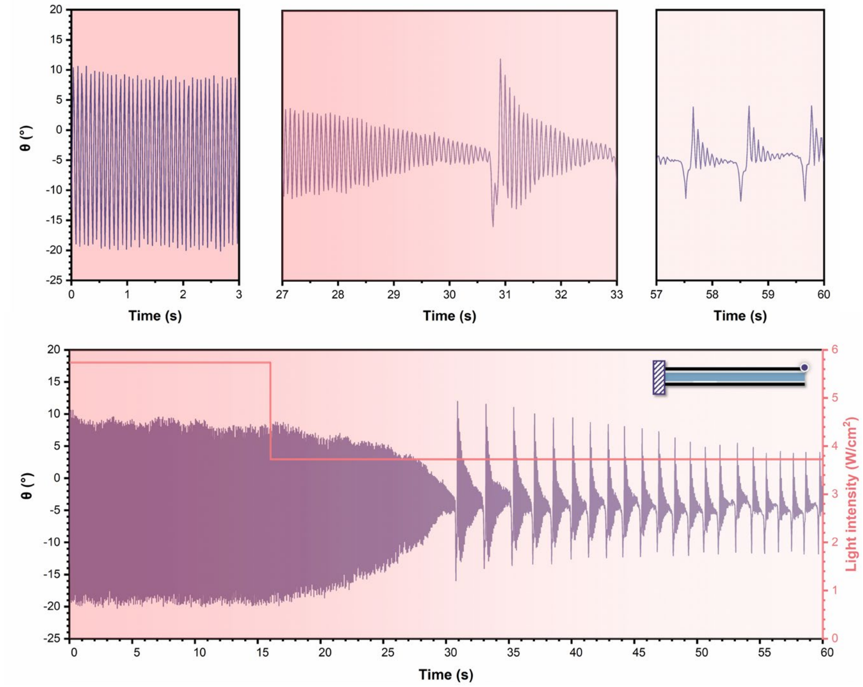

Fig. S16: The undulation-oscillation bifurcation under decreasing input. When the input dropped from 5.494 back to 3.728 W/cm^2, the motion would regulate from the oscillation to the undulation (Supplemetary Movie 3). Specifically, when the received energy was not enough to sustain the high-energy oscillation, the amplitude would decrease significantly, and the defect region could buckle again to tune the motion accordingly. After several cycles, the undulation trajectory would be restored to the initial state.

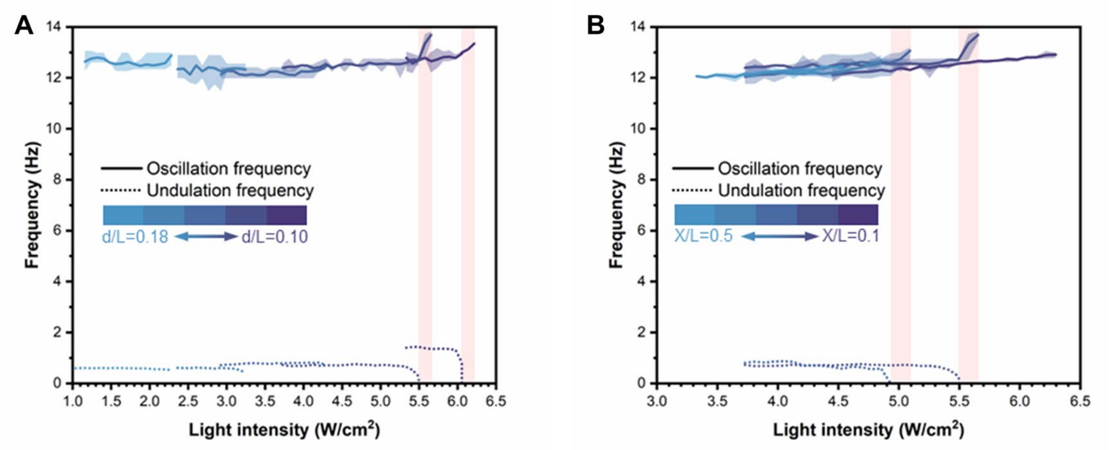

Fig. S17: (A) Frequency comparison of motions with different values of d/L when X/L was fixed at 0.20. (B) Frequency comparison of motions with different values of X/L when d/L was fixed at 0.12. The red regions refer to the oscillation after the transition. When X/L was fixed at 0.20, all the structures presented the buckling behavior with stable undulation and oscillation frequencies. The increasing d would slightly decrease the undulation frequency because the length of the defect region extended with more time for wave propagation. In contrast, when the length of the defect region was invariant, the transition only occurred for X/L in the range of 0.20 to 0.30. The undulation frequencies were stable and close, which indicated that the buckling frequency was more dependent on the d than the X. Besides, all the oscillation frequencies seemed more constant, which were mainly determined by the stiffness of the structure.

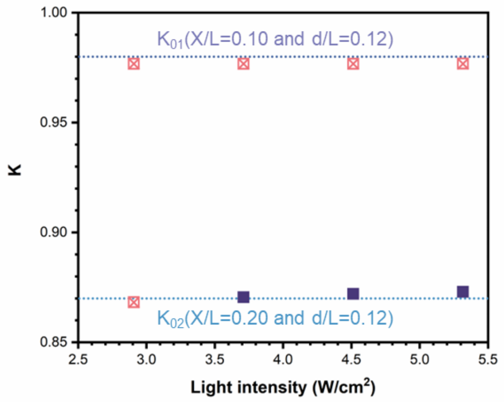

Fig. S18: Comparison of the relationship between K and K_0. The red hollow symbols represent states where $K < K_0$, indicating that the bending tendency dominates and the trilayer remains in the tracking or oscillation state. In contrast, the solid symbols indicate $K > K_0$, where the buckling tendency dominates, enabling the undulation. For X/L = 0.20 and d/L = 0.12, K can exceed the critical K_0 (blue dotted line), leading to the undulation. In contrast, for groups where K consistently remains below K_0 (e.g., X/L = 0.10 and d/L = 0.12; purple dotted line), the systems are limited to exhibiting only tracking and oscillation.

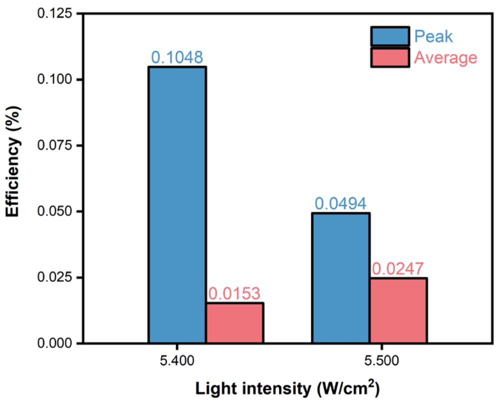

Fig. S19: Comparison of peak and average energy conversion efficiencies between undulation and oscillation modes. The total input energy was calculated as

$$E_{\text{input}} = I \times A \times t$$

where I was the light intensity, A was the illuminated area (a rectangular spot of 3.5 mm × 4.5 mm), and t = 1.1 s was chosen to ensure at least one full motion cycle (Fig. 3F). Accordingly, the peak efficiency (η_{peak}) was defined as

$$\eta_{\text{peak}} = \frac{\text{Max(Kinetic energy)}}{E_{\text{input}}}$$

and the average efficiency (η_{avg}) as

$$\eta_{\text{avg}} = \frac{\text{Ave(Kinetic energy)}}{E_{\text{input}}}$$

where the kinetic energy was calculated based on the simulated modeling results.

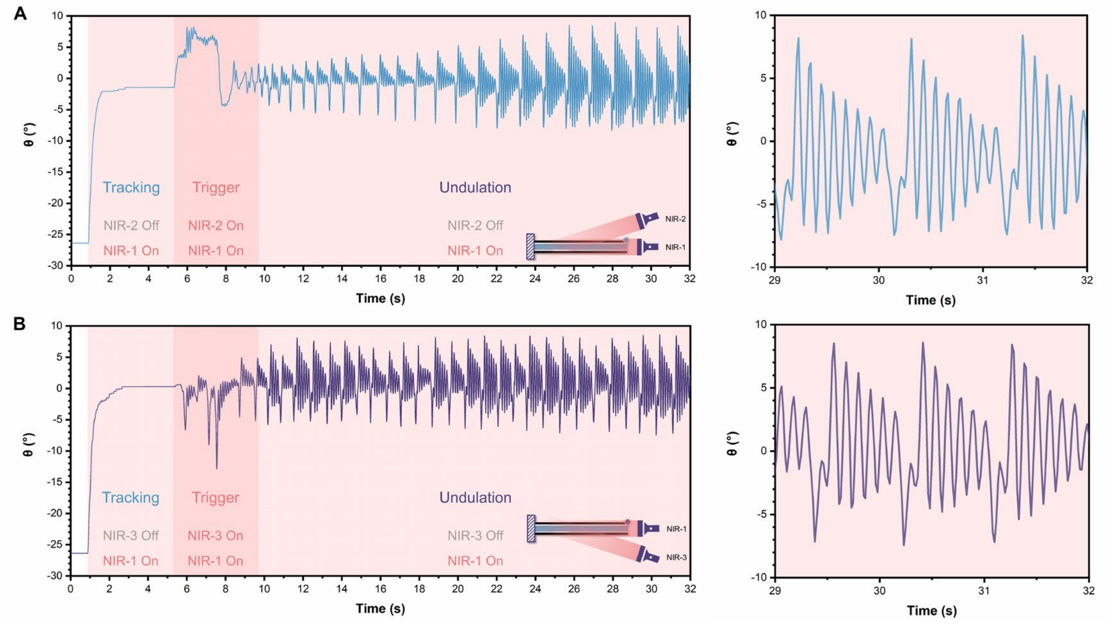

Fig. S20: Using a second laser to trigger the tracking-undulation bifurcation from different incidence directions. When the asymmetric trilayer was heated into the tracking state close to the tracking-undulation bifurcation point, additional energy introduced to the defect region would trigger the buckling. Either excited from (A) above or (B) below would result in successful undulation even after removing the second laser. The difference was that the entire transition would be accomplished rapidly from the below irradiation since the monolayer could generate higher stress immediately without heat transfer. In contrast, the transition from the above irradiation would be smoother if the trilayer utilized multiple buckling cycles to reach dynamic equilibrium step by step. Overall, two triggers contributed similar results in amplitudes and frequencies.

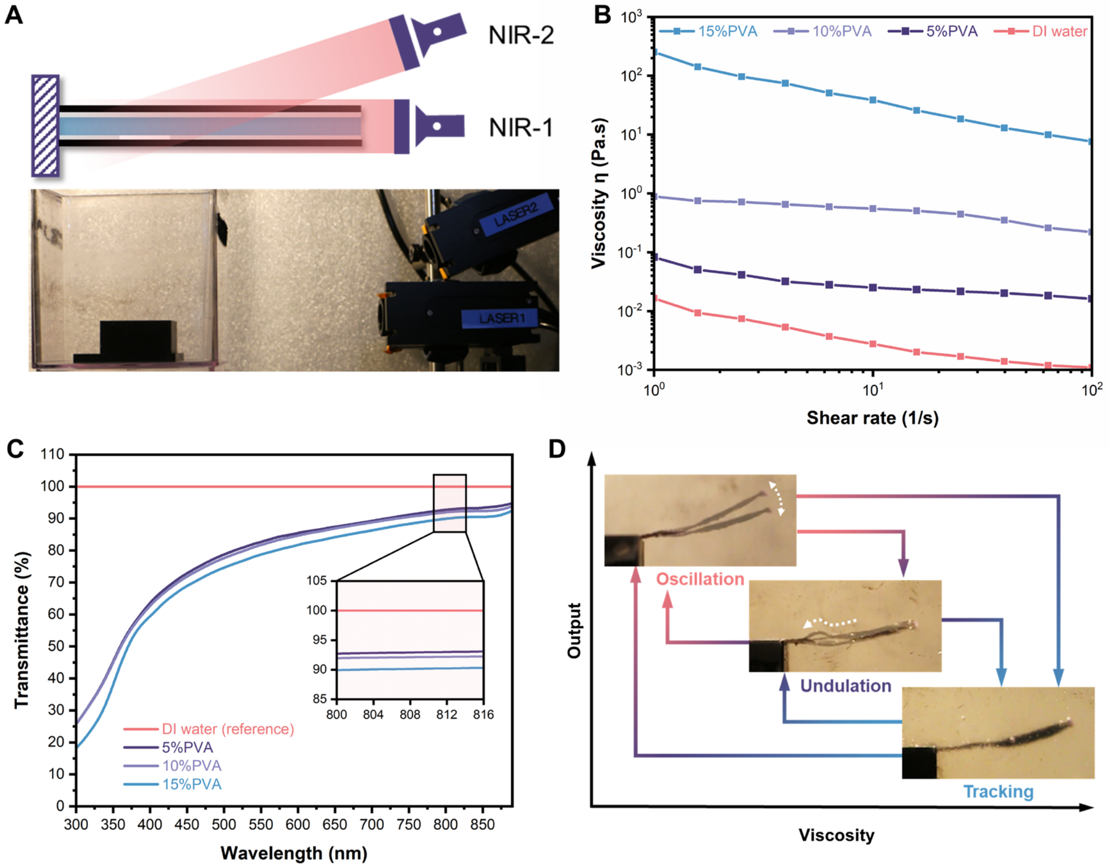

Fig. S21: Motion adaptation under fluid conditions. (A) We introduced a second laser to increase the energy input; otherwise, the trilayer could only achieve the tracking due to the rapid heat dissipation in the aqueous environment. The whole setup was fixed, followed by adding the prepared solution into the acrylic tank (see Section 1.8). (B) With the higher concentration of the PVA, the viscosity would dramatically rise by orders of magnitude from ~10^{-3} to 10^{1} Pa s. Therefore, the asymmetric trilayer required a greater portion of the energy expended to overcome the environmental resistance, leading to decreased system energy to maintain motion. As a result, under the same light intensity, the trilayer oscillated in water, self-adapted to undulate at low PVA concentrations, and eventually exhibited tracking at high concentrations. (C) The

transmittance of the solution to the laser was sufficiently high, above 90% in all cases. The input was high enough for underwater oscillation to neglect the effect of transmittance difference. (D) Motion switchability under fluid conditions with varying viscosity.

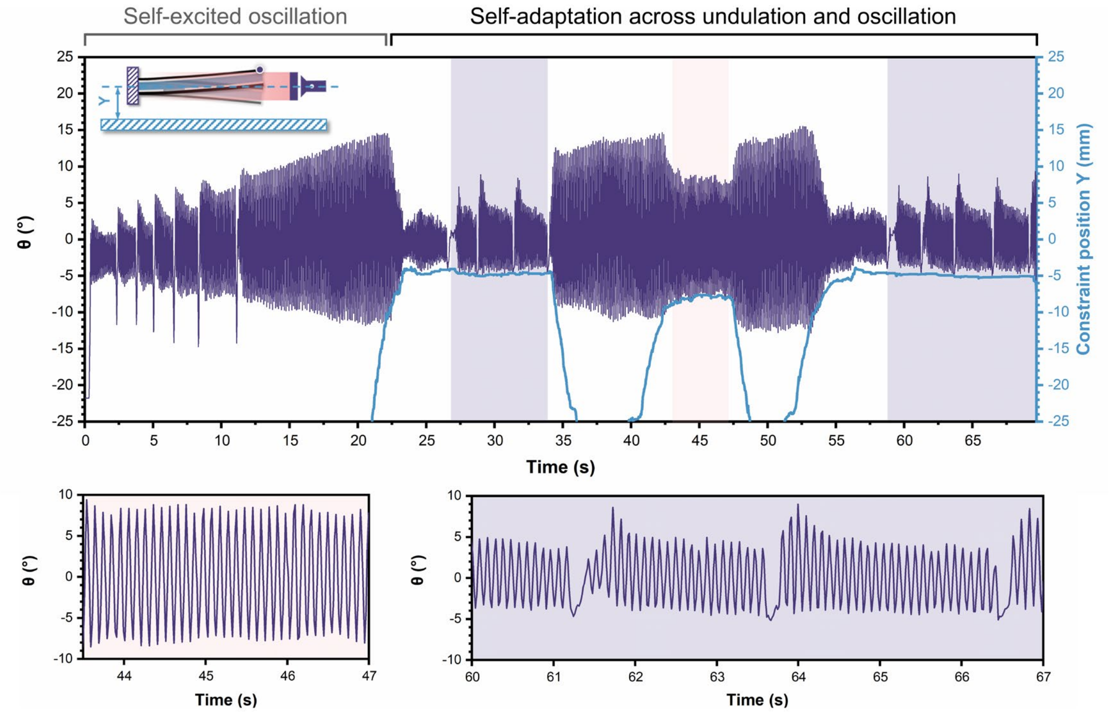

Fig. S22: The entire process of self-excitation and self-adaptation across the undulation and oscillation. With enough input, the asymmetric trilayer would be self-excited with amplifying amplitude by multiple buckling. Eventually, it would enter the oscillation state by passing through the undulation. However, when the physical constraint was applied, the soft actuator would dissipate partial energy by colliding with the solid surface. By tuning the constraint position, the dissipative energy could be controlled. Therefore, the motion behavior could be regulated autonomously, such as the pink region for oscillation with limited amplitude and the purple region for undulation.

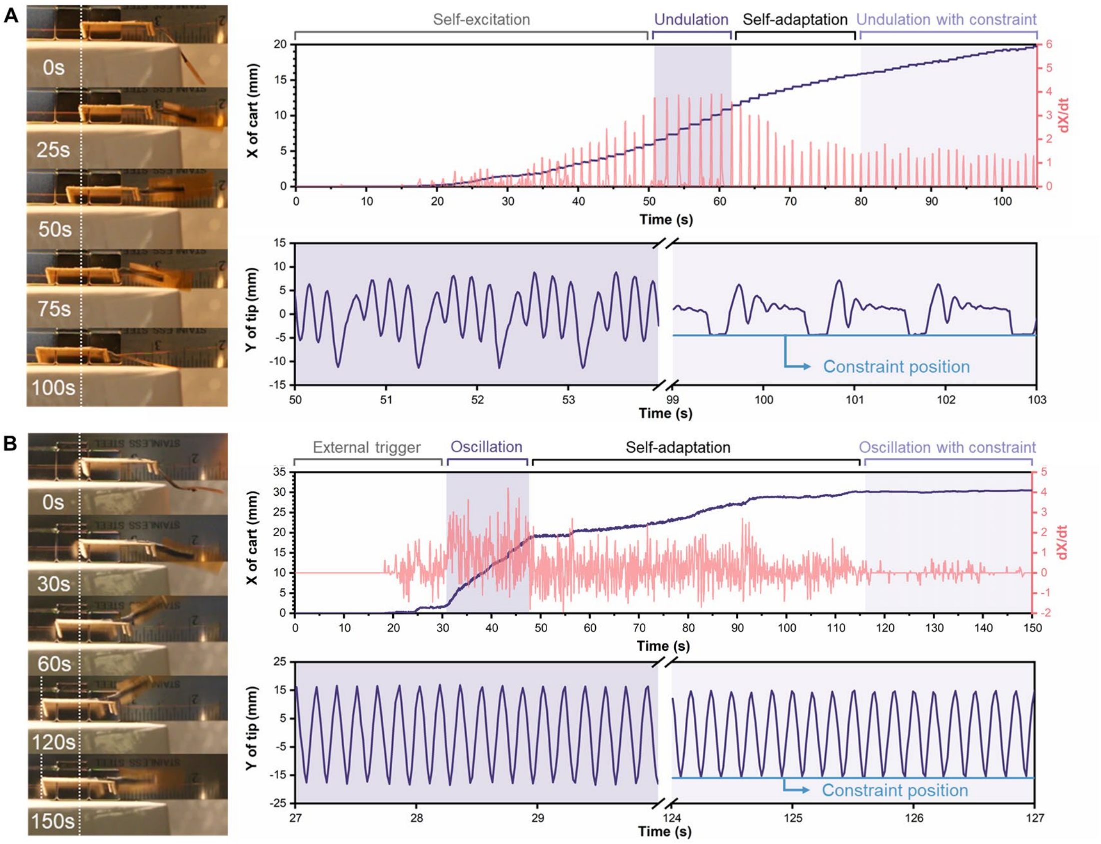

Fig. S23: Comparison of (A) asymmetric and (B) symmetric trilayer for cart demonstrations. (A) The asymmetric trilayer, following self-excitation—a capability absent in the symmetric trilayer—achieved self-continuous locomotion under a lower energy input (3.967 W/cm²), without being obstructed by the physical constraint (Supplementary Movie 10). (B) In contrast, under the same conditions, even with a higher light intensity (6.732 W/cm²), the cart equipped with the symmetric trilayer was blocked by the constraint, with most of the input energy dissipated through repeated contact with the constraint (Supplementary Movie 11).

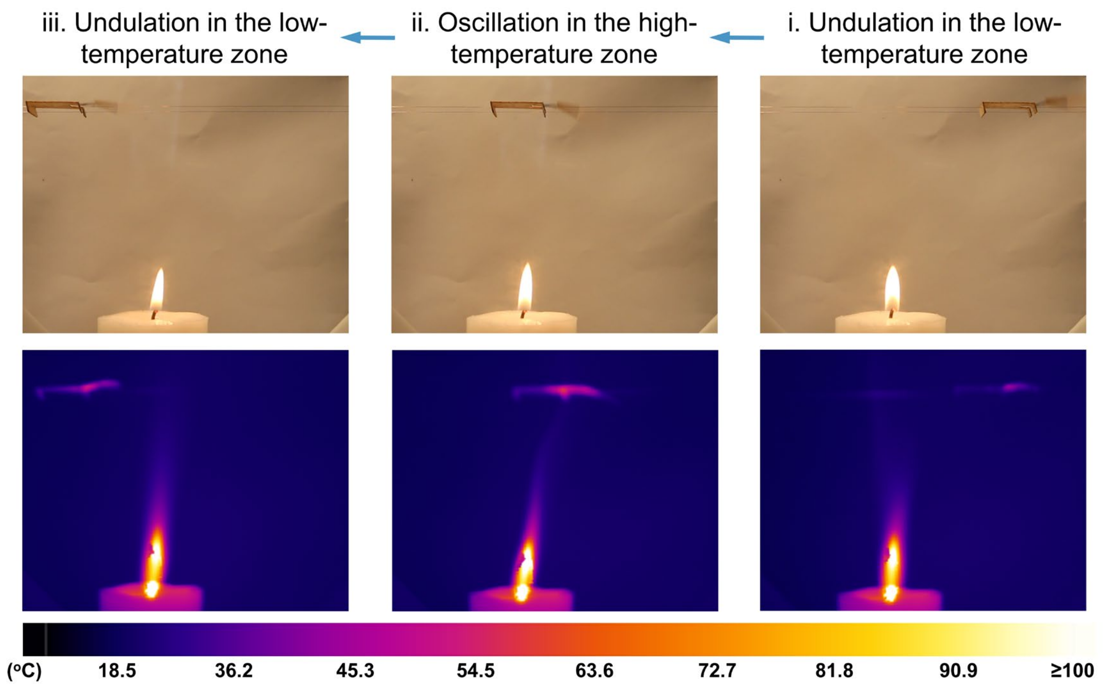

Fig. S24: Photo and IR camera imaging of the process of the cart passing through the high-temperature zone under constant light. The cart started from the right side (i) away from the high-temperature zone, passed through the high-temperature zone (ii), and reached the left side (iii) with the motion switching from undulation to oscillation and back to undulation, accordingly. The constant light was illuminated from the right side horizontally at the light intensity of 4.772 W/cm^2.

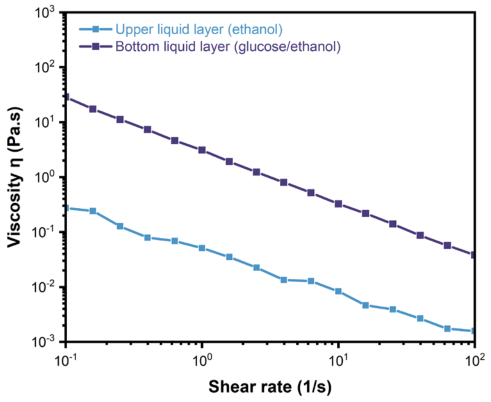

Fig. S25: Viscosity comparison of the upper and bottom liquid layers. The two layers of solution exhibited a significant difference in viscosity and can remain stably stratified for at least three days.

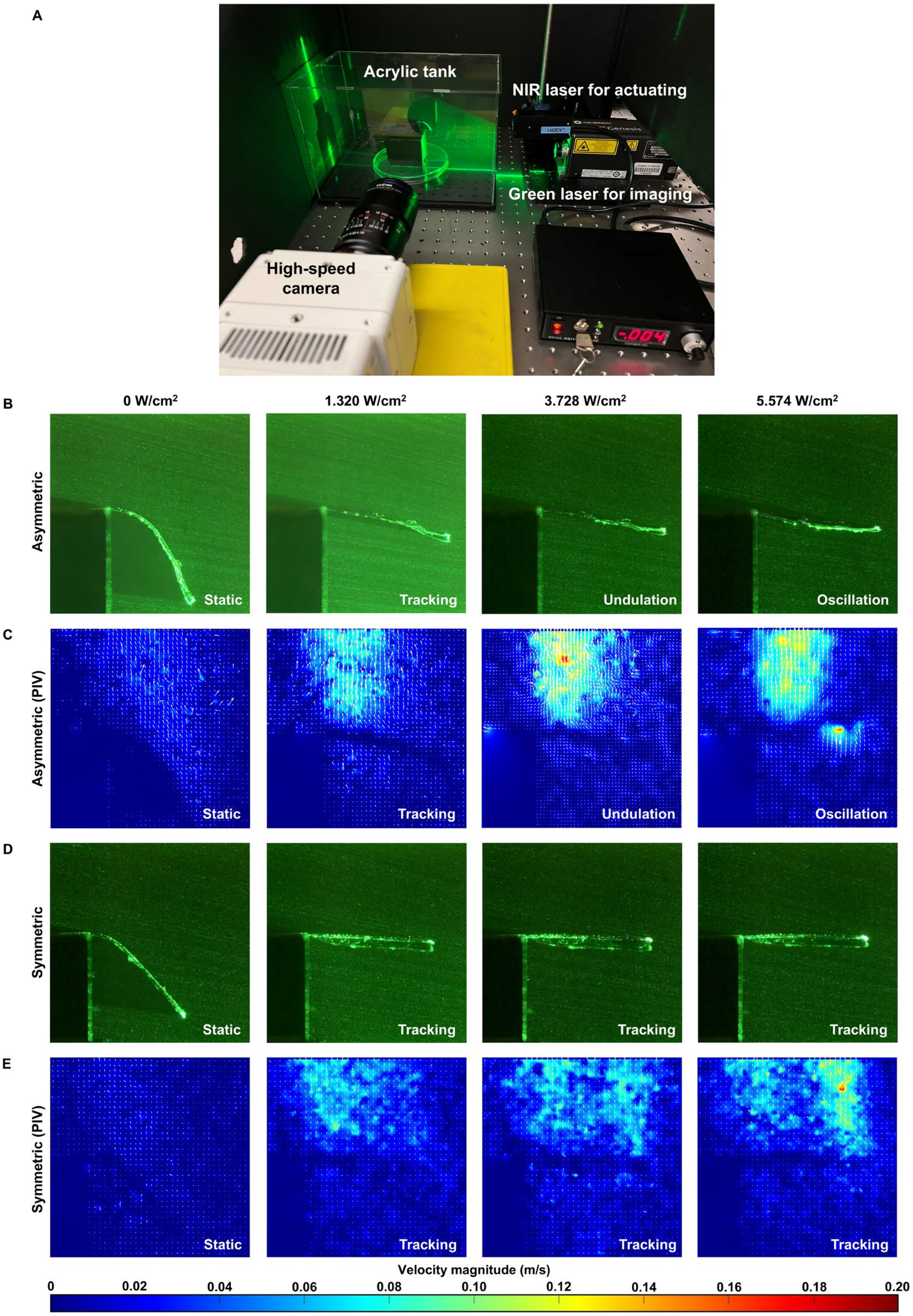

Fig. S26: Influence of different motions on surrounding airflow. (A) Two lasers were applied in the setup. In addition to the NIR laser for triggering and controlling motion, a green laser with low intensity was used to capture the real-time position of BEHS particles for imaging (see Section 1.10). (B) The various motion modes under different light intensities of the asymmetric trilayer: static (0 W/cm^2), tracking (1.320

W/cm^2), undulation (3.728 W/cm^2), and oscillation (5.574 W/cm^2). (C) The corresponding velocity analysis results indicated that the buckling enhanced the updraft airflow over the defect region, and increasing light intensity led to higher velocity due to greater buckling amplitude. On the other hand, a second hotspot occurred in the oscillation state, suggesting vortex formation around the tip (see Fig. S27). (D-E) The motions of symmetric trilayer under the same condition exhibited only the tracking, with the increased airflow velocity above the structure attributed to Brownian motion resulting from the enhanced photothermal effect of LCE [5].

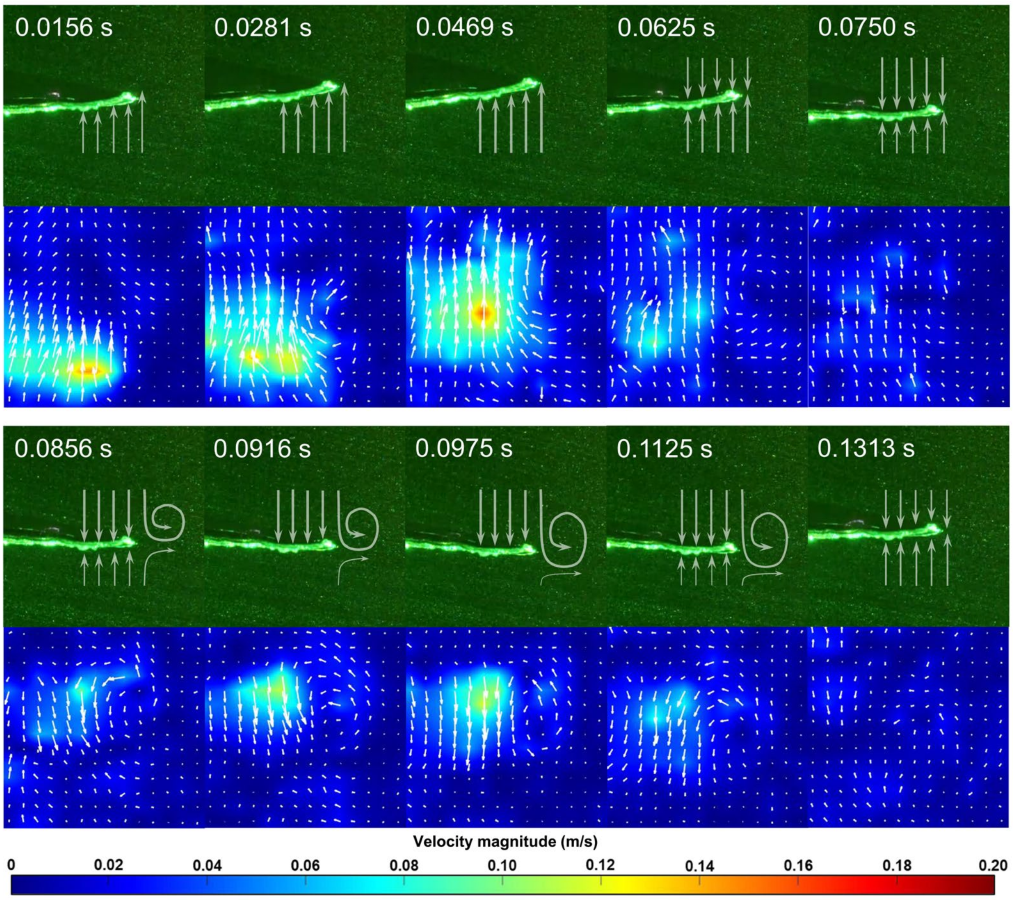

Fig. S27: Non-reciprocating vortex generated by the oscillating tip. When the actuator bent upward, a significant updraft was generated (0.0156 ~ 0.0469 s). Afterward, as the actuator moved downward from the highest point, the tip drove the nearby airflow downward synchronously (0.0625 ~ 0.1125 s). Meanwhile, the previous upward airflow persisted due to inertia. The convergence of these two airflow streams resulted in the formation of a counterclockwise vortex.

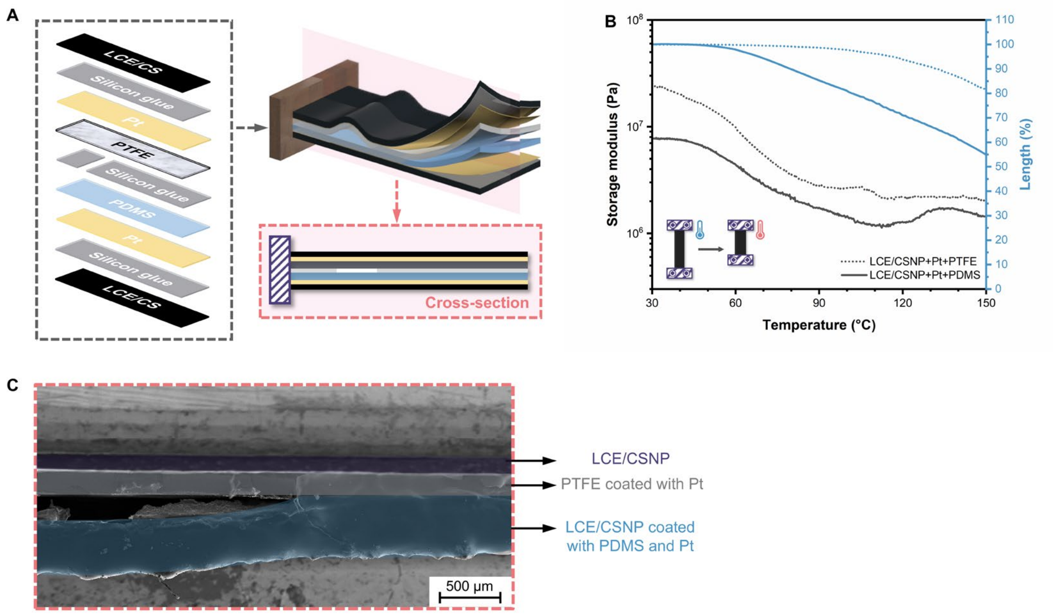

Fig. S28: Triboelectric nanogenerators (TENG) demonstrations based on the asymmetric structure. (A) Schematic of the asymmetric multilayer structure, with the interlayer being PTFE and PDMS coated with Pt. (B) Cross-sectional SEM images of the asymmetric multilayer structure. (C) The LCE/CSNP+Pt+PDMS demonstrated larger changes in modulus and length than the LCE/CSNP+Pt+PTFE under varying temperatures in the DMA test, which played a crucial role in generating the dynamic bow-shaped structure for instability-induced motions.

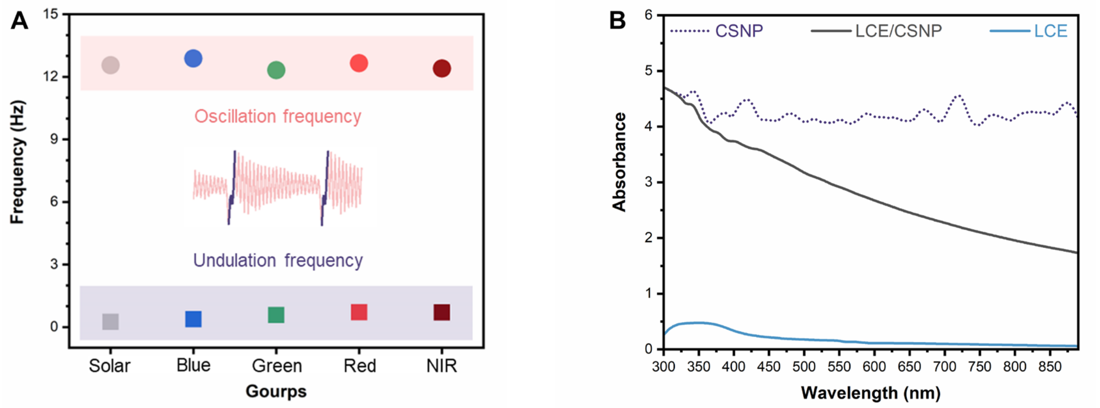

Fig. S29: Broad-spectrum operation of the asymmetric trilayer. (A) Instability-induced undulation could be extended to different types of light sources with distinct undulation and oscillation frequencies. (B) The CSNP-embedded LCE monolayer presented large absorbances (over 1.0) across from visible light to near-infrared light, which enabled the various remote triggers.

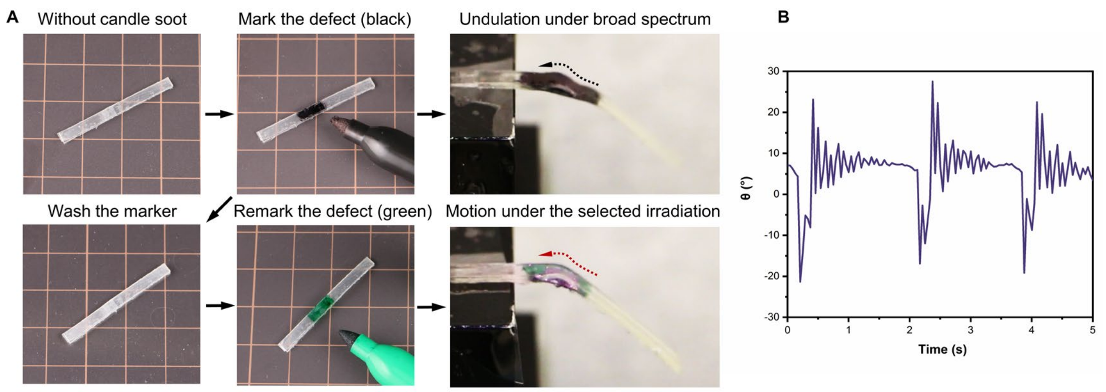

Fig. S30: The selective and erasable performance based on commercial markers. (A-B) By marking the defect with commercial black ink, as an alternative to the entire CSNP doping, the multimodal behavior can still be realized. Afterward, the black ink can be easily erased by ethanol and changed to others for the desired irradiation.

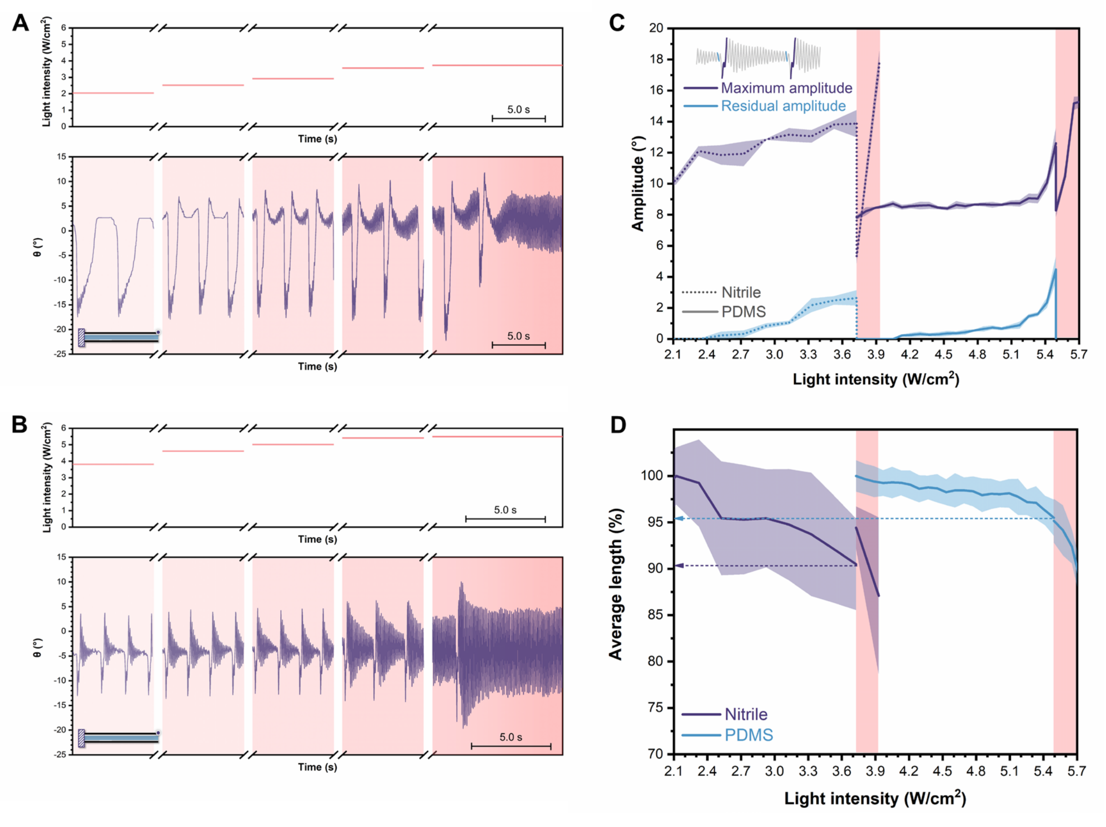

Fig. S31: Motion comparison of asymmetric trilayer structures with interlayer of Nitrile and PDMS. (A) With similar thermomechanical properties of Nitrile as the interlayer (Table S1 and S2), the asymmetric trilayer structure still demonstrated buckling behavior and undulation-oscillation bifurcation at the input of 3.728 W/cm^2. (B-C) Like the PDMS counterpart, as the input rose, the buckling would transform into oscillation eventually, which presented a similar change in the amplitude. (D) However, the nitrile-based trilayer was not as well supported as the PDMS counterpart, which resulted from the doubled contractions due to the thermoplastic behavior of Nitrile. Specifically, the modulus loss of Nitrile in high temperatures led to a larger shrinkage

of the system than that of PDMS, so the undulatory motion could not be maintained.

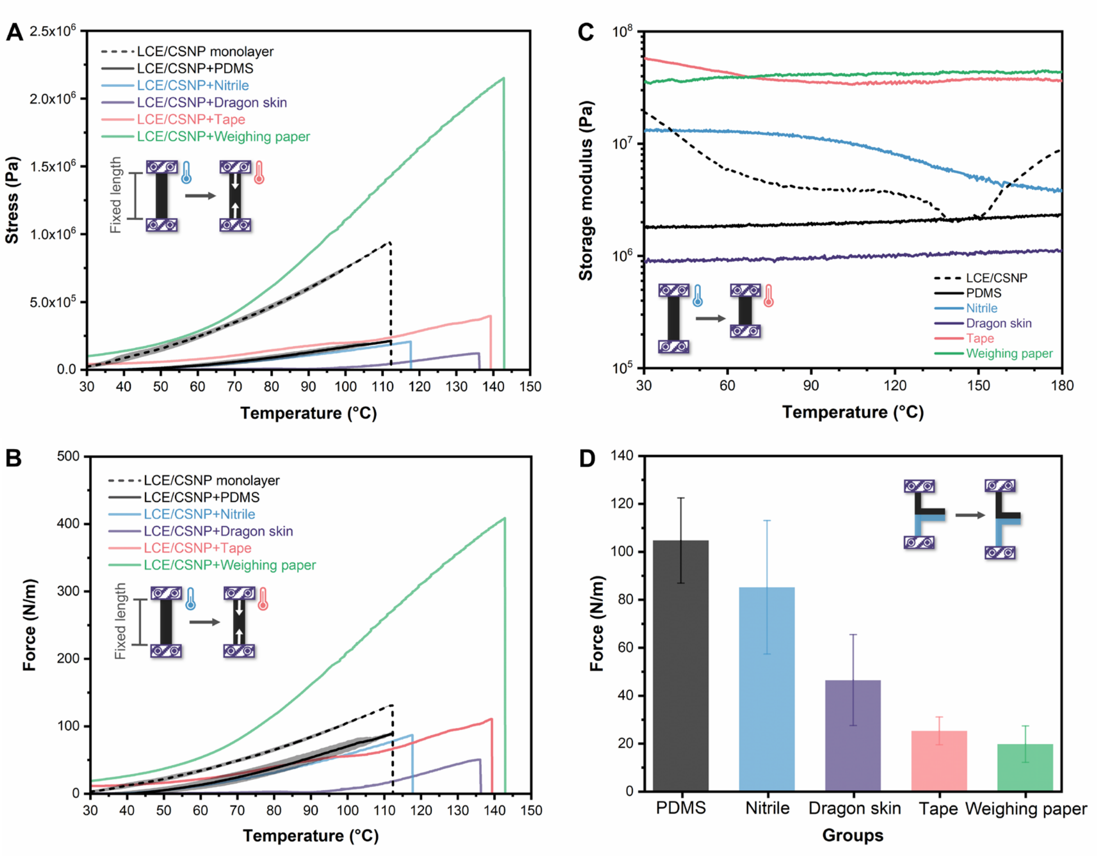

Fig. S32: Thermomechanical characterization of asymmetric trilayer structures with different interlayers. (A) Internal stresses, (B) internal forces, (C) moduli, and (D) adhesion would be vastly variant among different interlayer polymers. Since Nitrile showed the closest thermomechanical properties to the PDMS, the asymmetric trilayer fabricated in the same way achieved the transition from tracking to buckling, and finally oscillation.

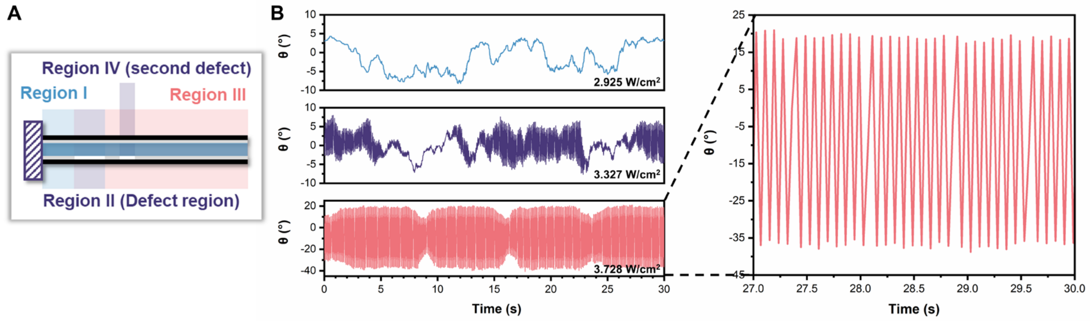

Fig. S33: Multimodal behavior of the asymmetric trilayer with two defect regions. (A) An additional defect (Region IV) was introduced within Region III. (B) The presence of the second defect affected all three motion modes and introduced an additional oscillation frequency in the oscillation mode.

Table S1. Motion comparison of asymmetric trilayer structures with different

interlayers

Interlayer	PDMS	Nitrile	Dragon skin	Tape	Weighing paper
Tracking	√	√	√	√	√
Buckling	√	√	×	×	×
Oscillating	√	√	√	×	×

Table S2. Differences of asymmetric trilayer structures with different interlayers under varying temperatures

Interlayer			PDMS	Nitrile	Dragon skin	Tape	Weighing paper
Δ*	ΔStress (Pa)	70 °C	292755	301003	345972	245054	-68954
		80 °C	377860	393412	460348	324411	-147151
		90 °C	473617	494633	595232	424573	-251018
		100 °C	585344	612229	735156	554219	-353838
	ΔForce (N/m)	70 °C	24.8448	28.3807	47.1958	19.5588	-30.6212
		80 °C	27.8860	34.4176	62.5307	25.4274	-51.3184
		90 °C	30.7162	39.5428	81.7946	34.7792	-77.7292
		100 °C	35.1412	46.4328	98.0623	49.8296	-104.854

Note: *Δ = Value of the LCE monolayer - Value of the bilayer

Table S3. Motion comparison of asymmetric trilayer structures with different layers

Trilayer	LCE+LCE+LCE	LCE+PDMS+PDMS	PDMS+PDMS+LCE
Initiation	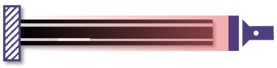	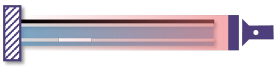	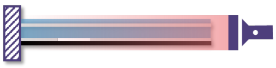
Equilibrium	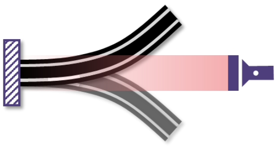	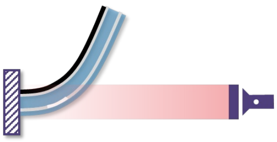	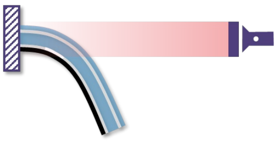
Optical photos	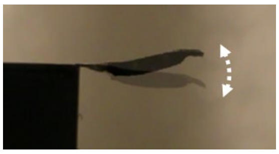	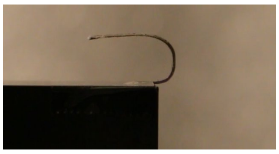	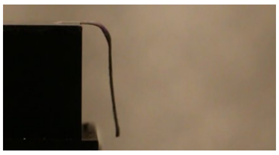

Note: When the actuating LCE/CSNP replaced the interlayer, the whole would contract

at the same ratio, behaving like a monolayer structure without buckling. Besides, if any actuating layer was replaced by the passive PDMS, the trilayer would perform like a bilayer, which could realize the positive feedback instead of the negative feedback to bend against the light.

References

1 Deng, X., Mammen, L., Butt, H.-J. & Vollmer, D. Candle Soot as a Template for a Transparent Robust Superamphiphobic Coating. *Science* **335**, 67-70, doi:10.1126/science.1207115 (2012).

2 Zhao, Y. *et al.* Sunlight-powered self-excited oscillators for sustainable autonomous soft robotics. *Science Robotics* **8**, eadf4753, doi:10.1126/scirobotics.adf4753 (2023).

3 Shi, P., Zhao, Y., Liu, Z. & He, X. Liquid crystal elastomer composite-based photo-oscillator for microrobots. *Journal of Composite Materials* **57**, 633-643, doi:10.1177/00219983221146618 (2023).

4 Zhao, Y. *et al.* Antagonistic-contracting high-power photo-oscillators for multifunctional actuations. *Nature Materials* **24**, 116-124, doi:10.1038/s41563-024-02035-3 (2025).

5 Uhlenbeck, G. E. & Ornstein, L. S. On the Theory of the Brownian Motion. *Physical Review* **36**, 823-841, doi:10.1103/PhysRev.36.823 (1930).
