# Supplementary material for: Bio-inspired multimodal soft actuator with environmental self-adaptation
Source: Nat Commun. 2025 Aug 15;16:7630. doi: 10.1038/s41467-025-62328-2 (PMC12356950; doi:10.1038/s41467-025-62328-2)
Supplement: Supplementary file 2 — Description of Additional Supplementary Files [file 41467_2025_62328_MOESM2_ESM.pdf]

<u>Description of Additional Supplementary Files</u>

Supplementary Movie 1. The autonomous self-sustained undulation and simulation. The asymmetric trilayer was fixed on the stage, and the NIR light was irradiated from the right side horizontally at the input of 3.728 W/cm^2. The experimental results were played in a real-time manner while the simulation results were played at 0.1 x speed.

Supplementary Movie 2. The motion adaptation and undulation-oscillation bifurcation under increasing input. The asymmetric trilayer was fixed on the stage, and the NIR light was irradiated from the right side horizontally, with the light intensity increased from 3.728 to 5.735 W/cm^2 step by step. The undulation-oscillation bifurcation occurred at the input of 5.494 W/cm^2. All motions were played in a real-time manner.

Supplementary Movie 3. The undulation-oscillation bifurcation under decreasing input. The asymmetric trilayer was fixed on the stage, and the NIR light was irradiated from the right side horizontally, with the light intensity decreased from 5.735 to 3.728 W/cm^2 directly. All motions were played in a real-time manner.

Supplementary Movie 4. Excitation comparison of asymmetric and symmetric actuators. Both actuators were fixed on the stage, and the NIR light was irradiated from the right side horizontally at the input of 5.735 W/cm^2. The asymmetric actuator achieved self-excitation, while the symmetric one required external force to oscillate after entering the tracking state. All motions were played at 2.0 x speed.

Supplementary Movie 5. Self-excitation with different inputs. The asymmetric actuator was fixed on the stage, and the NIR light was irradiated from the right side horizontally. With different inputs, the trilayer could spontaneously be excited into different dynamic equilibria, with 3.728 W/cm^2 for undulation and 5.735 W/cm^2 for oscillation. All motions were played in a real-time manner.

Supplementary Movie 6. Tracking-undulation bifurcation triggered from different incidence directions. The asymmetric actuator was fixed on the stage, and the first NIR light was irradiated from the right side horizontally at the input of 3.407 W/cm^2 only to achieve the tracking motion. Afterward, a second NIR light was directed to the defect region from above or below directions for approximately 5 s to trigger the bifurcation. All motions were played in a real-time manner.

Supplementary Movie 7. The underwater motion adaptation to the increasing input. The asymmetric actuator was fixed on the stage in the tank, and the first NIR light was irradiated from the right side horizontally at the constant input of 9.348 W/cm^2. In the meantime, a second NIR light was directed to the defect region, with the light intensity increased from 9.348 to 21.390 W/cm^2 step by step. The tracking-undulation and undulation-oscillation bifurcations occurred at the input of 11.355 and 19.383 W/cm^2 of the second laser, respectively. All motions were played at 2.0 x speed.

Supplementary Movie 8. Self-adaptation to varying fluid viscosity and temperature. The asymmetric actuator was fixed on the stage in the tank, with the first NIR light directed horizontally from the right side at a constant input of 9.348 W/cm^2. Meanwhile, a second NIR second was directed to the defect region at the input of 19.383 W/cm^2, providing sufficient energy to trigger the underwater oscillation. When the solution environment was altered with different concentrations of PVA, the actuator spontaneously adapted to various motions. Similarly, as the surrounding temperature increased, the system energy of the actuator rose, leading to the motion changes from tracking to undulation, and eventually oscillation. All motions were played in a real-time manner.

Supplementary Movie 9. Self-adaptation to the physical contact. The asymmetric actuator was fixed on the stage, and the NIR light was irradiated from the right side horizontally for constant oscillation. Afterward, the actuator made contact with a solid constraint (a glass slide) and was able to switch

between undulation and oscillation depending on the position of the constraint. All motions were played in a real-time manner.

Supplementary Movie 10. Self-adaptation of the cart to the physical contact. The asymmetric actuator of the cart was irradiated by the NIR light from the right side horizontally. The cart continued to move forward without being obstructed by physical contact with the solid constraint due to the self-adaptation. All motions were played at 5.0 x speed.

Supplementary Movie 11. Comparison of cart locomotion based on the asymmetric and symmetric actuators. Both two carts were constrained to the track irradiated by the NIR light from the right side horizontally but with different actuators. The input to trigger the asymmetric one was 3.967 W/cm^2, which was much lower than that for the symmetric one at 6.723 W/cm^2. All motions were played in a real-time manner.

Supplementary Movie 12. Motion mode self-adaptation of the cart passing through the high-temperature zone. The cart was started from the right side under the constant light illumination horizontally, and passed through the high-temperature zone to the left side eventually. During the process, the motion underwent the switching between undulation and oscillation. The whole process was played at 50.0 x speed while each motion was captured in a real-time manner.

Supplementary Movie 13. Motion mode self-adaptation of the cart passing through the liquid-liquid interface. The cart was started from the low viscosity zone under the constant light illumination parallel to the cart, and passed through the liquid-liquid interface to the high viscosity zone. During the process, the motion underwent the switching from oscillation to undulation. The whole process was played at 8.0 x speed while each motion was captured in a real-time manner.

Supplementary Movie 14. The influence of different motions on surrounding airflow. The asymmetric and symmetric actuators were fixed on the stage in the tank with a green laser for imaging and an NIR laser for actuating. All motions were played at 0.075 x speed.

Supplementary Movie 15. The motion adaptation of the asymmetric trilayer with a second defect. The asymmetric trilayer with two defects was fixed on the stage, and the NIR light was irradiated from the right side horizontally, with the light intensity increased from 2.925 to 3.728 W/cm^2 step by step. All motions were played in a real-time manner.
